# The Effects of Different Repetitive Transcranial Magnetic Stimulation (rTMS) Protocols on Cortical Gene Expression in a Rat Model of Cerebral Ischemic-Reperfusion Injury

**DOI:** 10.1371/journal.pone.0139892

**Published:** 2015-10-02

**Authors:** Milos R. Ljubisavljevic, Asma Javid, Joji Oommen, Khatija Parekh, Nico Nagelkerke, Safa Shehab, Thomas E. Adrian

**Affiliations:** 1 Department of Physiology, College of Medicine and Health Sciences, UAE University, Al Ain, UAE; 2 Malawi-Liverpool-Wellcome Trust Clinical Research Programme, Queen Elizabeth Central Hospital, College of Medicine, Blantyre, Malawi; 3 Department of Community Medicine, College of Medicine and Health Sciences, UAE University, Al Ain, UAE; 4 Department of Anatomy, College of Medicine and Health Sciences, UAE University, Al Ain, UAE; Fraunhofer Research Institution of Marine Biotechnology, GERMANY

## Abstract

Although repetitive Transcranial Magnetic Stimulation (rTMS) in treatment of stroke in humans has been explored over the past decade the data remain controversial in terms of optimal stimulation parameters and the mechanisms of rTMS long-term effects. This study aimed to explore the potential of different rTMS protocols to induce changes in gene expression in rat cortices after acute ischemic-reperfusion brain injury. The stroke was induced by middle cerebral artery occlusion (MCAO) with subsequent reperfusion. Changes in the expression of 96 genes were examined using low-density expression arrays after MCAO alone and after MCAO combined with 1Hz, 5Hz, continuous (cTBS) and intermittent (iTBS) theta-burst rTMS. rTMS over the lesioned hemisphere was given for two weeks (with a 2-day pause) in a single daily session and a total of 2400 pulses. MCAO alone induced significant upregulation in the expression of 44 genes and downregulation in 10. Two weeks of iTBS induced significant increase in the expression of 52 genes. There were no downregulated genes. 1Hz and 5Hz had no significant effects on gene expression, while cTBS effects were negligible. Upregulated genes included those involved in angiogenesis, inflammation, injury response and cellular repair, structural remodeling, neuroprotection, neurotransmission and neuronal plasticity. The results show that long-term rTMS in acute ischemic-reperfusion brain injury induces complex changes in gene expression that span multiple pathways, which generally promote the recovery. They also demonstrate that induced changes primarily depend on the rTMS frequency (1Hz and 5Hz vs. iTBS) and pattern (cTBS vs. iTBS). The results further underlines the premise that one of the benefits of rTMS application in stroke may be to prime the brain, enhancing its potential to cope with the injury and to rewire. This could further augment its potential to favorably respond to rehabilitation, and to restore some of the loss functions.

## Introduction

Transcranial Magnetic Stimulation (TMS) is a well-established, non-invasive technique that allows the assessment and modulation of brain excitability. Repetitive TMS (rTMS), a variant of TMS that involves repeated application of TMS pulses, may facilitate or suppress brain activity with variable behavioral effects. Research generally shows that the functional effects of rTMS on cortical excitability depend on stimulation intensity, frequency and the overall stimulation pattern. It appears that rTMS repeated at fixed high-frequency intervals (> 4 Hz) increase cortical excitability, while stimuli repeated at low-frequency (~ 1Hz) decrease it [1]. rTMS protocols utilizing patterned stimulation like theta-burst patterns (bursts of 3–5 pulses at 50–100 Hz, repeated at 5 Hz, i.e. theta rhythm) appear to enhance cortical excitability if applied continuously (cTBS), whereas if applied intermittently (iTBS) they tend to lower cortical excitability [2]. Furthermore, changes in cortical excitability elicited by rTMS may outlast the duration of the stimulation [3], a finding that has prompted considerable exploration of the potential of rTMS neurological and psychiatric therapy. Limited but promising data currently exist for the benefit of the rTMS in the treatment of depression, tinnitus, anxiety disorders, neurodegenerative diseases and pain syndromes [4]. In stroke, rTMS was also applied based on a model of interhemispheric competition for sensory and motor processing [5–7], prompting development of two conceptually different stimulation strategies [8,9]: one aiming to increase excitability of the affected hemisphere by excitatory rTMS [10–12] and the other aiming to suppress excitability of the unaffected hemisphere by inhibitory rTMS [13–16]. In patients with chronic post-stroke hemiparesis, for example, stimulation of the affected motor cortex with 5 Hz [17] or 10 Hz [10] facilitated practice-dependent plasticity and improved motor learning, whereas inhibition of the contra-lesional hemisphere with 1-Hz rTMS also enhanced motor recovery [13,14,17].

The current understanding of mechanisms aiding functional recovery after stroke suggest complex mechanisms that involve resolution of edema and necrotic tissue, reperfusion of the ischemic penumbra [18] and a set of neuronal compensatory mechanisms. These mechanisms include the recruitment of new/additional pathways, disinhibition of redundant neuronal connections and formation of new neural networks to take over function of the damaged areas [19]. What remains unclear are the effects of rTMS and how it interacts with such complex cellular and molecular milieu. In animals, rTMS increases the content of ATP and microtubule associated protein-2 expression [20] while promoting the recovery of the neuronal function [21], enhancing the long-term potentiation of the hippocampal neurons [22], preventing ischemic neural damage [23], enhancing anti-apoptotic mechanisms in the peri-ischemic area [24] and inducing neuroprotective effects [25].

However, in human studies, variable effects of rTMS interventions were reported [26,27]. The duration of the effects also seems to vary and depend on several factors, including the timing of the rTMS application (subacute or chronic stroke), the patient’s characteristics and the site of stimulation [28–30].The possibility of varying rTMS parameters (intensity, pattern, duration) makes the potential effects and therapeutic outcomes even more unpredictable. Furthermore, the effectiveness of rTMS may be influenced by the nature of the underlying pathological processes. A common assumption is that therapeutic effects in patients can be predicted based on the modulatory effects of rTMS in healthy subjects. However, there is no real evidence to suggest that this is always the case because the susceptibility to the conditioning effects of rTMS may well depend on the underlying pathology. Finally, it is apparent that the long-term clinical improvements caused by rTMS cannot be entirely explained by immediate electrophysiological processes caused by rTMS. Rather, processes beyond instantaneous electrophysiological modulation of neuronal activity related to adaptive changes in gene expression may be involved in sustaining rTMS effects.

This study primarily aimed to examine whether different rTMS protocols have differential effects on gene expression in lesioned cortices after ischemic-reperfusion brain injury. Thus, we examined the effects of four standard rTMS protocols (1Hz, 5 Hz, cTBS and iTBS) on functional recovery and changes in gene expression in lesioned rat cortices with subacute cerebral ischemic-reperfusion injury induced by middle cerebral artery occlusion (MCAO). We assessed changes in the expression of 98 genes known to be altered by stroke, as well as those potentially involved in promoting recovery after stroke, with real-time RT-PCR after two weeks of rTMS.

## Methodology

### Ethics statement

All experiments and procedures were carried out according to the National Institute of Health (NIH) guidelines for the care and use of laboratory animals and approved by the appropriate local or national ethics board (permit number A06/11 from the Animal Ethics Committee of the College of Medicine and Health Sciences, UAE University). All animals were singly housed in cages under standard conditions (12 hour light-day cycle) with free access to water and food before and after all procedures. All efforts were made to minimize animal suffering and to reduce the number of animals used.

### Animals and experimental groups

A total of 149 male Wistar rats weighing 260–270 g were used for this study. Out of 104 animals that underwent middle cerebral artery occlusion-reperfusion (MCAO), 87 rats survived, providing a survival rate of 84%. Excluded from the experiment were rats not showing hemiplegia, neurological deficits (at 48 hours, see later behavioral testing) or a clearly visible infarcted area two weeks after MCAO (n = 6). Consequently, 81 rats were randomly assigned to the following nine MCAO groups (n = 9 animals/group): four rTMS MCAO groups (1 Hz, 5Hz, cTBS and iTBS stimulation groups), four rTMS MCAO sham-stimulated groups (ShSTIM) using the same rTMS protocols, and one MCAO group. In addition, 18 animals were used in control (CON) (n = 9) and sham-operated (ShMCAO) group (n = 9). The remaining animals (n = 27) were used to assess the stroke volume, as will be discussed later.

### MCAO surgery

We used a unilateral middle cerebral artery occlusion (MCAO) model of brain infarction described in previous studies, with minor modifications. Anesthesia was administrated as an intramuscular injection, a combination of ketamine (110 mg/kg) and xylazine (10mg/kg) (Troy Laboratories, NSW, Australia). When necessary, this anesthesia was followed with maintenance doses depending on the reflex withdrawal response and breathing rate. Artificial tears ointment was applied to the animals’ eyes for protection and lubrication. Using a homoeothermic blanket control unit and feedback-regulated heating lamps, the animals’ temperature was monitored and maintained between 37 and 37.5°C during and immediately after the surgical procedure. A midline neck incision of the skin was performed to reveal the left common, external (ECA) and internal (ICA) carotid artery. A 4–0 monofilament nylon suture with a silicon coated tip (Doccol Co-operation, Redlands, CA, USA) was introduced through a small incision in ECA and advanced into the ICA occluding the origin of the middle cerebral artery. After 90 minutes of occlusion, the monofilament was removed and the ECA permanently tied. The animals were then placed in individual cages and carefully monitored until they started drinking and eating spontaneously. Sham-operated animals were treated similarly except for the MCAO.

### Behavioral testing

**Fig 1:**
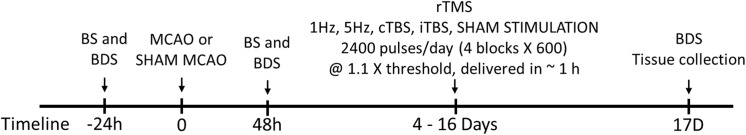
Timeline of the MCAO-rTMS experiments (BS—5-point neurological deficit Bederson scale, BDS–Behavioral Deficit Score).

Functional deficits after MCAO and rTMS were assessed by rating the severity of deficits with a series of simple neurological tests combined into a composite Behavior Deficit Score (BDS) [31]. BDS included walking initiation, spontaneous circling, rotation, righting, tail hang, paw placement, horizontal bar and postural reflexes. Each test was scored on an ordinal scale, based on a set of pre-determined criteria described elsewhere [31–33] with total BDS ranging from 0–21, where 0 indicates fully expressed deficit and 21 corresponds to normal animal. In order to reduce variance and increase data reliability the tests were repeated twice to three times, depending on the test [34]. Functional deficit was also scored on a modified 0-5-point Bederson scale (BS) [35]. BS expresses the degree of gross neurological impairment on an ordinal scale, where 0 indicates no deficit, 1 indicates mild forelimb weakness, 2 indicates severe forelimb weakness and consistent rotation to the side of deficit when lifted by tail, 3 indicates spontaneous circling or walking to the contralateral side, 4 indicates walking only when stimulated or depressed level of consciousness, and 5 signifies animal unresponsive to stimulation. MCAO animals that received a score of 0 (i.e. no deficit) at 48 hours post-occlusion were excluded from the study. Behavioral changes were evaluated 24 hours before (-24h) and 48 hours after MCAO (48h), as well as after the last rTMS session 17 days after MCAO (17d) (see Fig 1). The examiner performing behavioral testing was blinded for the stimulation (real vs. sham) and MCAO (real vs. sham-operated).

### Stroke assessment

To assess the size of lesioned area and the consistency of the stroke size brains from randomly selected animals with MCAO (n = 6) were stained 48 hours later using triphenyl tetrazolium chloride (TTC) stain. In addition, TTC staining was used to confirm the absence of lesion in sham-operated animals (n = 3). To assess the effects of rTMS on the size of lesioned area additional 14 animals were randomly assigned into MCAO (n = 7) and iTBS (n = 7) group and their brains stained on day 17.

For TTC staining, the animals were guillotined, the brains were dissected out and 2mm coronal slices were cut using a brain matrix (Braintree Scientific, MA; USA). The slices were then stained with 2% TTC (Sigma Inc.), dissolved in saline for 30 min at 37° C. The slices were then fixed in 10% formalin overnight. Each section was scanned using a stereomicroscope (Leica MZ16A, Heerbrugg; Switzerland), and the size of the pale injured area versus deep red color non-injured area was marked and calculated using the indirect method [36] (Leica Application Suite v 3.0, Heerbrugg; Switzerland).

To further confirm the character of the MCAO immunohistochemistry for parvalbumin, calbindin and calretinin was performed on free floating sections (n = 4). Initially, rats were perfused using 4% paraformaldehyde 2 hours after termination of rTMS. Brains were cut coronally at 70μm thickness using a vibratome. Brains sections were incubated with polyclonal rabbit anti-parvalbumin (Cat #PV-25; Swant, Switzerland; 1:20,000), polyclonal rabbit anti-calbindin (Cat# D-28K; Swant, Switzerland; 1:20,000) and polyclonal rabbit anti-calretinin (Cat# 7699/3H; Swant, Switzerland; 1:10,000) overnight. After rinsing in phosphate buffered saline PBS, the sections were incubated in biotinylated goat anti-rabbit IgG (1:500) for 1 hour then in extravidin perioxidase conjugate (Sigma, 1:1000) for another hour. To visualize immunoreactivity the sections were incubated for 5 minutes in a solution of 25 mg diaminobenzidine (DAB) in 50 ml 0.1 M phosphate buffer (PB, pH 7.4) with 7.5μl hydrogen peroxidase (30%) and 1 ml nickel chloride (3.5%) added to intensify the reaction. Finally the sections were rinsed in PB and mounted on gelatin coated slides. Later sections were dehydrated in alcohol, cleared in xylene and mounted with DPX. All antibodies were diluted in PBS containing 0.3% triton.

### rTMS and experimental groups

Four rTMS protocols were used: 1Hz, 5Hz, cTBS and iTBS. During the stimulation, rats were un-anesthetized, wrapped with their eyes partly covered with a thick cloth and gently restrained on a platform with Velcro straps. To reduce stress related to handling, restraining, and stimulation, rats were familiarized to these conditions (including rTMS noise) one week prior to surgery.

rTMS was started 3 days after MCAO ischemic injury (Fig 1). Rats were stimulated once per day, between 9 and 11 am, for 10 days (two weeks with a 2-day break) using the standard figure-of-eight coil (MagPro Magnetic Stimulator, Farum, Denmark), positioned and oriented to induce synaptic activity in most cortical areas of the left hemisphere [37]. Initially, the motor threshold was grossly estimated by adjusting the stimulation intensity for each animal so that it barely evoked twitches of hind limbs and body muscles [38]. Thereafter, the stimulation was increased by 10%. The mean stimulus strength was 30.0±1.5% of maximal stimulator output strength (range 28–32%). The motor threshold was randomly checked at the beginning of the stimulation session for each animal. There were no significant changes in thresholds between stimulated groups during the course of the experiment. The selected stimulation intensity did not induce any visible discomfort to the animals; they remained generally restful throughout the experimental sessions.

The animals in each rTMS group received 2400 pulses per day. The number of pulses was chosen to mimic, as closely as possible, the stimulation rTMS protocols used in human studies. To exclude the potential effects of duration, the daily stimulation protocol was divided into four blocks, with 600 pulses/block. The time between the blocks was adjusted to keep the total duration of stimulation between 50 and 55 min for all protocols. In 1 Hz rTMS protocol four 10 minute blocks, with a four-minute pause in-between were given, while in 5 Hz rTMS protocol a continuous 2 min stimulation repeated at 15 min intervals was given. In iTBS, four blocks of ten 50 Hz bursts (3 pulses each), repeated 20 times at 5Hz intervals (600 stimuli), were given at 15 min intervals. For cTBS, the same burst (3 stimuli at 50 Hz, repeated at 5 Hz intervals) was given continuously in 20 s train, repeated four times at 15 min intervals. For sham-stimulated groups, we used the same stimulation protocols except that the coil was placed 15 cm above the rat’s head. For more information on rTMS protocols and their immediate effects on excitability in normal human brains, see Huag et al. (2005, 2009) and Fitzgerald et al. (2005) [1,2,39].

### Low-density gene expression array

Animals were guillotined and brain tissue was immediately placed in RNAlater (AM7021, Applied Biosystems, Carlsbad, CA, USA) and kept overnight at 4°C to allow thorough penetration of the tissue. Subsequently, a block of tissue a few millimeters thick was taken after the brain was coronally transected with a scalpel. The area from which the tissue was taken was determined according to each animal’s individual distribution of infarction. This area was easily detectable two weeks after MCAO, as the infarcted area had a clearly different color (whitened) from the rest of the tissue. Typically, the samples included the peri-infarct area (outer borders of the infarction also containing some healthy tissue), and a small portion of the infarcted area. We also sampled brain tissue from the contralateral hemisphere homologous to the infarct area and the peri-infarct area. Tissue samples were then frozen at -80°C pending further processing. Isolation of total RNA from the tissue was performed using the SV Total RNA Isolation System (Promega, Madison, WI; USA) according to the manufacturer’s instructions. We determined the concentration and purity of the RNA samples by measuring the absorbance at 260 nm (A260) and the ratio of the absorbance at 260 and 280 nm (ND-1000 NanoDrop). We performed gene expression analysis using custom pre-loaded Taqman Low Density Arrays (TLDA) (Format 96a, Applied Biosystems, Foster City, CA; USA) under universal cycling conditions (10 min 95°C, 40 cycles of 15 s at 95°C and 60 s at 60°C). We loaded assays on the TLDA panel with cDNA samples from each individual animal. Each port on the TLDA was loaded with 50μl of cDNA at a concentration of (4ng/μl) and 50μl of 2X Master Mix. Table 1 shows the target genes surveyed in the plates. We selected the reference gene by examining the representative samples from each group. The 18S gene showed the highest stability of expression across groups (mean cycle number of 11.85±0.42). We selected a calibrator from the sample with the lowest Ct value, from the control sample for control vs. MCAO comparison and from the MCAO sample for MCAO vs. rTMS treatment comparison. Data were analyzed using the comparative CT (2^-ΔΔCt^) method with a relative quantification RQmin/RQmax confidence set at 95% [40].

**Table 1:** Fold-changes in mRNAs (RQs) of all genes that showed expression on the microarray in MCAO and ShMCAO group using the control group as a calibrator. C and Sh in the group significance column indicate significant difference to CON and ShMCAO group, respectively.

Gene Symbol	Gene name	Assay ID	MCAO	Group Sig. Diff.	ShMCAO	Variance	Statistic (F)	df1	df2	Sig.
Actb	Actin beta	Rn00667869_m1	1.50	C Sh	0.92	0.011	12.219	2	13.727	0.001
Adcy1	Adenylate cyclase 1	Rn02115682_s1	0.64	C Sh	0.98	0.975	4.651	2	14.583	0.027
Adcy8	Adenylate cyclase 8 (brain)	Rn00567592_m1	0.76		0.88	0.002	0.650	2	12.238	0.539
Adm	Adrenomedullin	Rn00562327_m1	14.74	C Sh	0.95	0.001	18.484	2	12.441	0.0001
Adrb1	Adrenergic beta-1-receptor	Rn00824536_s1	0.68	C	1	0.209	5.783	2	13.468	0.015
Ak1	Adenylate kinase 1	Rn00577377_m1	1.1		0.9	0.060	0.736	2	13.699	0.497
Angpt1	Angiopoietin 1	Rn00585552_m1	8.08	C Sh	0.85	0.302	86.168	2	13.892	0.0001
Angpt2	Angiopoietin 2	Rn01756774_m1	26.01	C Sh	0.84	0.003	30.380	2	12.088	0.0001
Apoe	Apolipoprotein E	Rn00593680_m1	4.5	C Sh	0.93	0.000	22.946	2	12.610	0.0001
App	Amyloid beta (A4) precursor protein	Rn00570673_m1	1.22		0.94	0.213	1.827	2	13.822	0.198
Arrb1	Arrestin beta 1	Rn00563760_m1	1.09		0.93	0.110	0.771	2	13.942	0.481
Arrb2	Arrestin beta 2	Rn00563775_m1	1.58		1.02	0.188	2.290	2	13.452	0.139
Atf3	Activating transcription factor 3	Rn00563784_m1	24	C Sh	1.25	0.007	53.455	2	13.380	0.0001
Bag3	Bcl2-associated athanogene 3	Rn01754954_m1	3.5	C Sh	0.92	0.001	19.470	2	13.281	0.0001
Bai1	Rct59893.0	Rn01504966_m1	0.68	Sh	0.97	0.349	4.276	2	14.350	0.035
Bdnf	Brain-derived neurotrophic factor	Rn00560868_m1	0.39	C	1.01	0.027	6.287	2	9.858	0.017
Btg2	BTG family member 2	Rn00568504_m1	0.92		0.98	0.368	0.058	2	10.887	0.944
Car11	Carbonic anhydrase 11	Rn00598344_m1	1.03		0.93	0.018	0.351	2	13.847	0.710
Cck	Cholecystokinin	Rn00563215_m1	1.31		0.96	0.193	1.690	2	13.402	0.222
Cdk5	Cyclin-dependent kinase 5	Rn00590045_m1	1.33		0.92	0.061	2.775	2	12.533	0.101
Cited2	Cbp/p300-interacting transactivator	Rn00586705_m1	1.33	C	1	0.546	4.766	2	14.130	0.026
Clock	Clock homolog (mouse)	Rn00573120_m1	1.08		0.91	0.014	0.858	2	13.731	0.445
Cnr1	Cannabinoid receptor 1 (brain)	Rn00562880_m1	1.34		0.91	0.857	1.846	2	14.576	0.193
Creb1	Camp responsive element binding protein 1	Rn00578826_m1	1.51	C Sh	0.94	0.256	6.939	2	14.287	0.008
Cryab	Crystallin alpha B	Rn00564026_m1	2.07	C Sh	1.01	0.502	17.617	2	13.981	0.0001
Csnk1e	Casein kinase 1 epsilon	Rn00581130_m1	1.47	C Sh	0.93	0.122	7.520	2	12.963	0.007
Cxcl12	Chemokine (C-X-C motif) ligand 12	Rn00573260_m1	6.22	C Sh	0.93	0.001	18.006	2	13.010	0.0001
Cxcr4	Chemokine (C-X-C motif) receptor 4	Rn01483207_m1	7.63	C Sh	0.88	0.001	17.944	2	12.105	0.0001
Dagla	Diacylglycerol lipase alpha	Rn01454304_m1	0.87		0.99	0.623	0.281	2	14.363	0.759
Dbh	Dopamine beta-hydroxylase	Rn00565819_m1	0.53	C Sh	0.97	0.007	9.552	2	11.551	0.004
Ddit4	DNA-damage-inducible transcript 4	Rn01433735_g1	1.37	C	1	0.700	5.335	2	14.364	0.019
Dnaja1	Dnaj (Hsp40) homolog subfamily A	Rn00576012_m1	1.3	Sh	0.90	0.001	5.781	2	13.511	0.015
Drd2	Dopamine receptor 2	Rn01418275_m1	0.42		0.98	0.144	3.866	2	10.660	0.055
Dusp1	Dual specificity phosphatase 1	Rn00678341_g1	0.51	C Sh	1.02	0.175	8.473	2	14.242	0.004
Egr1	Early growth response 1	Rn00561138_m1	0.57	C Sh	1.11	0.812	9.374	2	13.905	0.003
Eng	Endoglin	Rn01438763_m1	3.32	C Sh	1.02	0.025	19.435	2	13.210	0.0001
Epn2	Epsin 2	Rn00573059_m1	1.56	C Sh	0.95	0.512	10.339	2	14.306	0.002
Faah	Fatty acid amide hydrolase	Rn00577086_m1	0.91		0.97	0.759	0.173	2	14.362	0.842
Fgf2	Fibroblast growth factor 2	Rn00570809_m1	2.06	C Sh	0.94	0.523	29.098	2	14.232	0.0001
Fos	FBJ osteosarcoma oncogene	Rn02396759_m1	0.34	C Sh	1.09	0.001	27.985	2	11.022	0.0001
Gabbr1	Gamma-aminobutyric acid (GABA) B receptor 1	Rn00578911_m1	1.11		0.98	0.768	0.708	2	14.522	0.509
Gad1	Glutamate decarboxylase 1	Rn00690300_m1	1.22		0.94	0.319	1.502	2	13.964	0.257
Gad2	Glutamate decarboxylase 2	Rn00561244_m1	1.11		0.9	0.025	0.978	2	13.791	0.401
Gadd45b	Growth arrest and DNA-damage-inducible	Rn01452530_g1	1.79	C	1.67	0.306	15.568	2	13.916	0.0001
Gapdh	Glyceraldehyde-3-phosphate dehydrogenase	Rn01775763_g1	0.92		0.89	0.010	0.125	2	13.561	0.884
Gfap	Glial fibrillary acidic protein	Rn00566603_m1	14.56	C Sh	0.95	0.002	51.164	2	12.655	0.0001
Gls	Glutaminase	Rn00561285_m1	1.46	Sh	0.85	0.038	5.336	2	13.834	0.019
Gnmt	Glycine N-methyltransferase	Rn00567215_m1	2.07	C Sh	0.77	0.700	7.724	2	14.462	0.005
Gpx1	Glutathione peroxidase 1	Rn00577994_g1	4.34	C Sh	0.92	0.002	15.431	2	12.630	0.0001
Gpx2	Glutathione peroxidase 2	Rn00822100_gH	6.14	C Sh	0.87	0.007	8.303	2	13.333	0.005
Gria1	Glutamate receptor ionotropic AMPA 1	Rn00709588_m1	1.07		0.94	0.305	0.401	2	13.553	0.677
Gria2	Glutamate receptor ionotropic AMPA 2	Rn00568514_m1	0.82		0.9	0.022	0.468	2	12.949	0.636
Gria3	Glutamate receptor ionotrophic AMPA 3	Rn00583547_m1	1.15		0.85	0.006	1.487	2	13.644	0.261
Gria4	Glutamate receptor ionotrophic AMPA 4	Rn00568544_m1	1.05		0.86	0.073	0.406	2	13.482	0.674
Grin2a	Glutamate receptor ionotropic NMDA2A	Rn01424654_m1	0.73		0.95	0.110	3.112	2	13.641	0.077
Grin2c	Glutamate receptor ionotropic N-methyl D-aspartate 2C	Rn00561359_m1	1.29	Sh	0.93	0.029	4.460	2	13.883	0.032
Hif1a	Hypoxia-inducible factor 1 alpha subunit	Rn00577560_m1	1.75	C Sh	0.87	0.012	21.840	2	13.125	0.0001
Hmox1	Heme oxygenase 1	Rn00561387_m1	17.62	C Sh	0.97	0.004	97.867	2	13.148	0.0001
Hspa4	Heat shock protein 4	Rn00596544_m1	1.79	C Sh	0.97	0.124	11.270	2	14.233	0.001
Hspd1	Heat shock protein 1	Rn00821037_g1	1.47	Sh	0.87	0.246	3.891	2	13.776	0.046
Hsph1	Heat shock 105/110 protein 1	Rn01412930_m1	1.03		0.89	0.011	0.561	2	13.668	0.583
Irf1	Interferon regulatory factor 1	Rn00561424_m1	12.89	C Sh	0.93	0.025	53.003	2	11.807	0.0001
Jun	Jun oncogene	Rn00572991_s1	0.25	C Sh	0.98	0.060	58.108	2	9.951	0.0001
Junb	Jun B proto-oncogene	Rn01251660_s1	5.44	C Sh	1.08	0.021	53.484	2	13.243	0.0001
LOC246295	Glycine- glutamate- thienylcyclohexylpiperidine-binding protein	Rn00595357_m1	1.38	Sh	0.93	0.060	5.062	2	14.004	0.022
Mapk1	Mitogen activated protein kinase 1	Rn00671828_m1	1.17		0.9	0.001	2.375	2	12.216	0.135
Mgll	Monoglyceride lipase	Rn00593297_m1	1.30		1	0.852	2.540	2	14.556	0.113
Mmp19	Matrix metallopeptidase 19	Rn01756324_m1	34.16	C Sh	0.9	0.098	515.110	2	11.774	0.0001
Mmp2	Matrix metallopeptidase 2	Rn01538170_m1	14.69	C Sh	0.89	0.005	23.448	2	12.486	0.0001
Mmp3	Matrix metallopeptidase 3	Rn00591740_m1	2.63	C Sh	0.92	0.765	15.743	2	14.195	0.0001
Mmp9	Matrix metallopeptidase 9	Rn00579162_m1	0.98		0.87	0.082	0.207	2	13.822	0.815
Myc	Myelocytomatosis oncogene	Rn00561507_m1	7.30	C Sh	0.93	0.016	36.603	2	11.794	0.0001
Napepld	N-acyl phosphatidylethanolamine phospholipase D	Rn01786262_m1	1.54	Sh	0.86	0.096	4.406	2	14.123	0.033
Nog	Noggin	Rn01467399_s1	1.09		0.89	0.006	1.460	2	13.229	0.267
Nos1	Nitric oxide synthase 1 neuronal	Rn00583793_m1	1.19		0.98	0.759	1.378	2	14.584	0.283
Nptx2	Neuronal pentraxin 2	Rn01756377_m1	0.63	C	1.03	0.213	5.060	2	13.938	0.022
Per3	Period homolog 3 (Drosophila)	Rn00709499_m1	1.91	C Sh	0.88	0.032	8.803	2	13.696	0.003
Plat	Plasminogen activator tissue	Rn00565767_m1	2.00	C Sh	1.03	0.130	9.461	2	13.679	0.003
Pxmp4	Peroxisomal membrane protein 4	Rn00597183_m1	1.39	C Sh	0.97	0.771	5.119	2	14.557	0.021
Rdx	Radixin	Rn01766742_m1	1.78	Sh	0.84	0.001	11.055	2	12.924	0.002
Slc3a1	Solute carrier family 3 member 1	Rn00568087_m1	3.82	C Sh	1.06	0.003	7.509	2	12.070	0.008
Tacr1	Tachykinin receptor 1	Rn00562004_m1	0.95		0.98	0.808	0.038	2	14.501	0.963
Tgfa	Transforming growth factor alpha	Rn00446234_m1	1.65	C Sh	0.98	0.929	10.059	2	14.658	0.002
Tnf	Tumor necrosis factor	Rn99999017_m1	5.71	C Sh	0.86	0.001	14.610	2	12.499	0.001
Trpv1	Transient receptor potential cation channel subfamily V	Rn00583117_m1	1.42		0.92	0.116	1.039	2	12.134	0.383
Tubb5	Tubulin beta 5	Rn00597407_m1	0.96		0.93	0.061	0.069	2	13.227	0.933
Vegfa	Vascular endothelial growth factor A	Rn00582935_m1	1.01		0.91	0.009	0.293	2	13.496	0.751

### Quantitative PCR

To confirm changes seen on low density arrays, we analyzed changes in expression of selected single genes by quantitative PCR (qPCR) using TaqMan gene expression assays (Applied Biosystems, Carlsbad, CA). We performed the reactions according to standard protocols, using the following gene expression assays: Gria2, BDNF and Gabbr1. We used 18S rRNA as a control, and ran each cDNA sample (n = 4/gene) in triplicate. Control wells with no template were included on each plate to check for contamination. Data were analyzed as described above using the comparative CT (2^-ΔΔCt^) method.

### Statistics

Data were evaluated for normality. Physiological data were analyzed by mixed factor ANOVA. The difference in infarct volume between MCAO and iTBS treated animals was analyzed using two-tailed unpaired student’s *t*-test. The difference in neurologic deficits on 5-point interval scale (BS) at 48h, between groups with and without MCAO were evaluated using Kruskal-Wallis H test. Changes in neurological deficits assessed by BDS score as categorical variable were analyzed in two nested trials using regression (analysis of covariance). The first trial sought to study the effects of MCAO on BDS before stimulation at 48h. For this BDS scores were regressed on MCAO, and their corresponding baseline values at -24h. The second trial aimed to examine the effects of rTMS on BDS after stimulation, i.e. at 17d. For this BDS at 17d were regressed on the categorical variable stimulus and their corresponding “baseline” value at 48h (only for groups with MCAO). Post-hoc comparison was done using Tukey's method in order to adjust for multiple comparisons.

We separated the statistical analysis of changes in gene expression (RQ values) into two parts, one comparing changes between the control, stroke and sham-stroke groups and one comparing changes between the stroke, rTMS and sham-stimulated groups. All changes in RQ values for all groups were initially examined for outliers; if present, they were excluded. We applied a one-way Welch ANOVA (robust test of equality of means) because in all but two of the examined genes, the assumption of homogeneity of variances was violated (Levene's Test of Homogeneity of Variance *p*<0.05) and because the RQ values for the majority of genes were not normally distributed (Shapiro-Wilk test of normality *p*<0.05). To rigorously control for multiple comparisons, the Welch ANOVA was followed by a Games-Howell post-hoc test for multiple comparisons. Changes were considered significant if *p*<0.05. In order to reduce the number of groups for comparison, the gene expression data and the BS and BDS data on functional deficits from all sham-stimulated groups were combined into a single sham-stimulated group.

## Results

### Physiological data

All results are given as the mean ± SEM. There were no significant differences (mixed factor ANOVA *p*<0.05) between the ShMCAO, MCAO, ShSTIM and rTMS groups during surgery (rectal temperature of ShMCAO = 36.8±0.1°C, MCAO = 36.7±0.15°C, ShSTIM = 36.9±0.15°C, and all rTMS groups = 36.8±0.15°C) or during the recovery period (rectal temperature of ShMCAO = 36.6±0.15°C, MCAO = 36.5±0.15°C, ShSTIM = 36.6±0.15°C, and all rTMS groups = 36.6±0.1°C).

### Assessment of the stroke and the effect of iTBS on stroke volume

**Fig 2:**
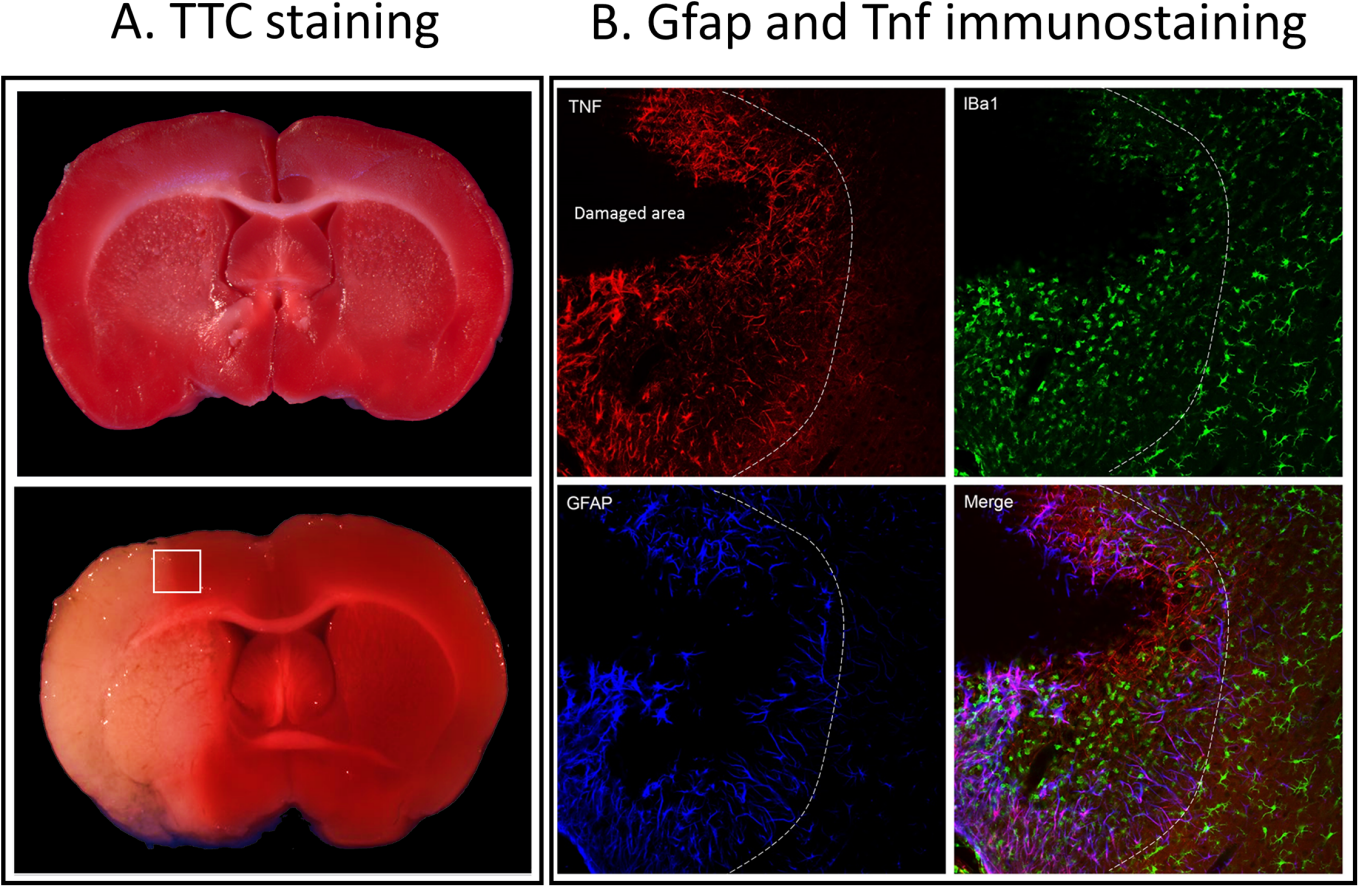
Photographs of TTC staining (A) showing sham-operated (upper panel) and MCAO brain (lower panel), and immune-staining (B) of Gfap and Tnf in the cerebral cortex from MCAO animal. The inset in the lower panel (A) indicates the typical region of the cortical tissue sampled for gene analysis corresponding also to Gfap and Tnf immune-staining.

On average the stroke involved 41.2% (range 40.3–44.6%, n = 6) of the left hemisphere total volume at 48h. TTC staining revealed significant brain damage that included the entire MCA territory in the parietal cortex. The injured areas typically involved cortices (frontal, parietal, temporal and parts of the occipital), striatum and parts of the thalamus and hypothalamus of the left hemisphere (Fig 2A). There was no visible lesion in sham-operated animals (n = 3).

Immunostaining was performed to further verify the MCAO and was in accordance with previously reported results. In brief, immunostaining of sections of the contralateral non-ischemic hemisphere showed no Tnf immunoreactivity whereas Iba1- positive cells showed ramified morphology of non-activated microglia and astrocytes with Gfap positive processes. In comparison, on the lesioned side the Tnf immunoreactivity was remarkably up-regulated with activated astrocytes and microglia in the peri-infarct zone (Fig 2B). The triple immunofluorescence labeling showed that Tnf immunoreactive processes were also Gfap positive (marker of astrocytes) but none of them were Iba1 positive (marker of microglia) confirming that Tnf was up-regulated in astrocytes and not in microglia.

Finally, to assess the effects of rTMS on the size of the infarcted area, in a separate group of animals (n = 14), brains were TTC stained after two weeks of iTBS (17d) and compared to MCAO only group. On average, at 17d the infarcted area involved 43.8% (range 41.3–46.6%, n = 7) of the left hemisphere’s total volume in MCAO animals. iTBS applied for two weeks had no significant effect on infarct size (*p*>0.05). The average infarct size in the iTBS group was 40.9% (range 39.4–44.1%, n = 7).

### The effect of MCAO and rTMS on behavior

**Fig 3:**
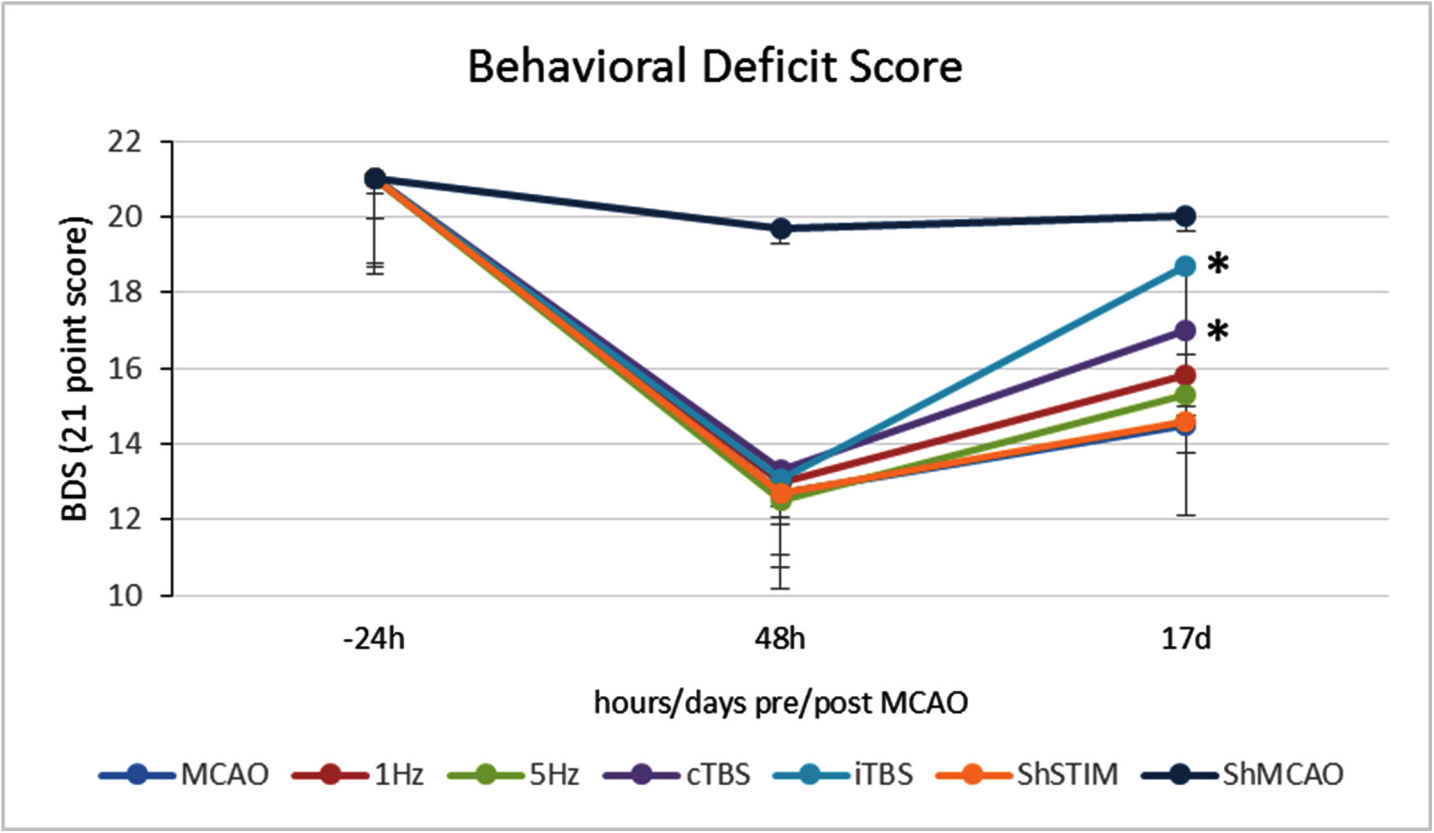
Changes in the behavior deficit score (BDS), on a 0-21point neurologic deficit scale where 0 indicates fully expressed deficit and 21 corresponds to normal animal, in MCAO, ShMCAO, 1 Hz, 5 Hz, cTBS, iTBS and ShSTIM groups prior to MCAO (-24h), 48 hours after MCAO (48h) and two-weeks later (17d). *p<0.0005 compared to MCAO group.

In terms of the 5-point neurological scale (BS) there was a significant change in median score in MCAO groups (1Hz, 5Hz, cTBS, iTBS, ShSTIM) compared to groups without MCAO (CON and ShMCAO) at 48h (*p<*0.0005 Kruskal-Wallis H test). The median BS was 3 in MCAO group, while in non-MCAO group it was 0. Similarly, the dependence of BDS on baseline values at 48h was highly significant (*p*<0.0005). MCAO reduced the median 21 point neurologic deficit score (BDS) by 6.8 points at 48 hours (Fig 3). The second trial analysis of BDS at 17 days, showed highly significant effect of rTMS (F_(1,19.153)_ = 2201.023, *p*<0.0005). cTBS and iTBS performed significantly better, improving BDS in stimulated groups, compared to MCAO, by 2.45 (*p*<0.0005) and 4.5 (*p*<0.0005) points, respectively.

### Changes in gene expression following MCAO

**Fig 4:**
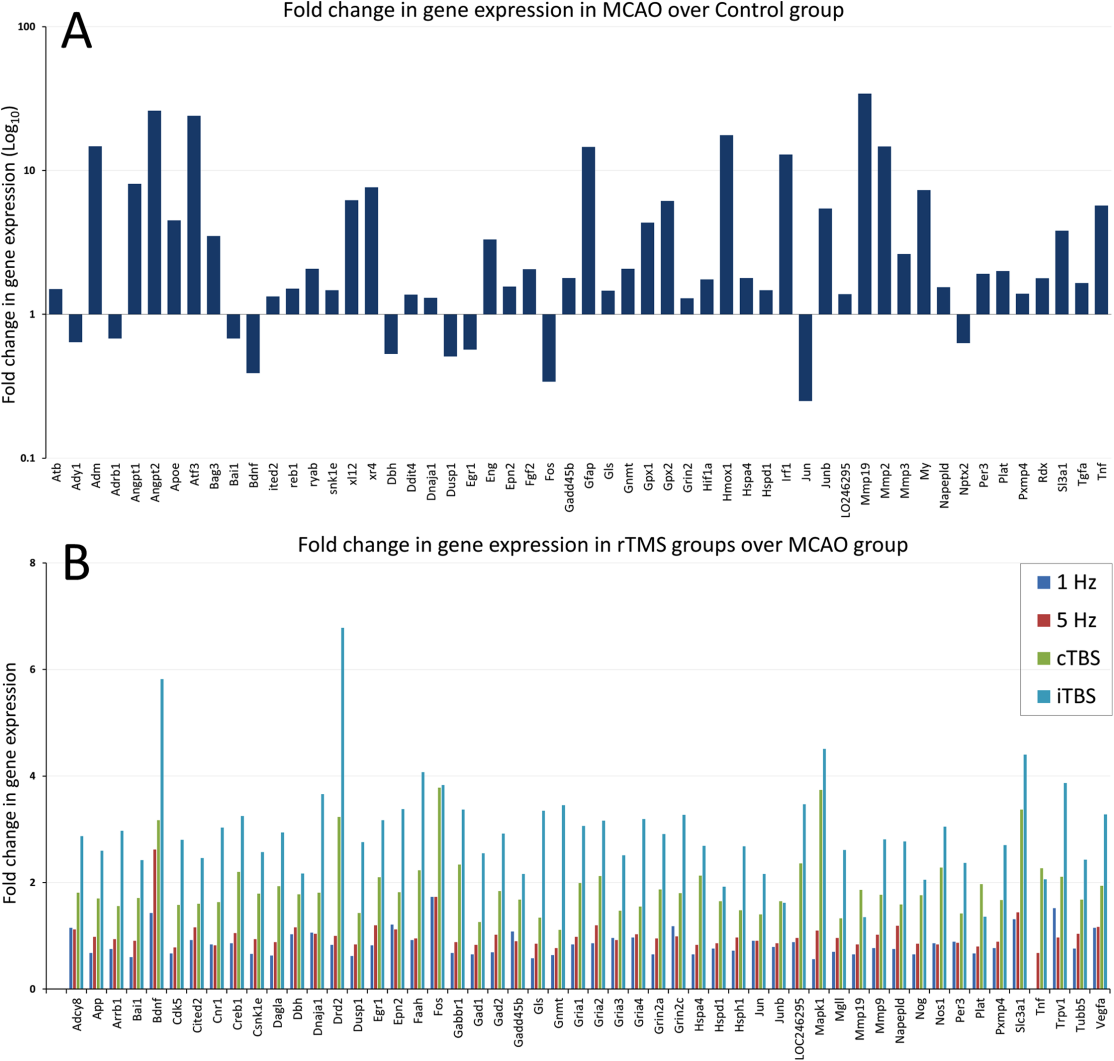
Fold-changes in mRNAs (RQs) of all genes that showed significant changes in expression after MCAO using the control group (CON) as a calibrator (A) and of all genes that showed significant changes in expression after two weeks of rTMS using the MCAO group as a calibrator (B).

The initial analysis compared changes between the stroke and sham-operated groups using the control group as a calibrator. Out of the 96 genes, nine were not amplified and were excluded from the analysis. MCAO induced significant change in the expression of 54 genes, out of which 10 were significantly downregulated, and 44 significantly upregulated (Fig 4A).

We conducted a one-way ANOVA to determine if MCAO induced changes in expression of genes. We initially assessed the data for outliers by inspecting the boxplot, and we assessed the normality through a Shapiro-Wilk test. For all genes, in all three groups, the expression was normally distributed, except for the following genes: Atf3, Btg2, Gadd45b, Irf1 in the ShMCAO group, as well as Drd2 in both control and ShMCAO group (Shapiro-Wilk test, *p*<0.05). Table 1 illustrates the homogeneity of variances for each gene, as assessed by Levene's test of homogeneity of variance. Considering that the assumption of homogeneity of variances was violated for several genes, we conducted a one-way Welch ANOVA to determine if gene expression differed between the groups. Group differences were examined by Games-Howell post-hoc test for multiple comparisons (see Table 1, group difference column). In this study, changes in gene expression were comparable to those reported earlier [41–43]. Highly-regulated genes were those related to inflammation, response to injury, brain ischemia, and regulation of angiogenesis, while no significant changes were found among genes related to neurotransmission and plasticity. The highly upregulated genes included Adm (15-fold), Angpt2 (26-fold), Atf3 (24-fold), Cxcr4 (8-fold), Gfap (15-fold), Gpx2 (6-fold), Hmox1 (17-fold), Irf1 (13-fold), Junb (5-fold), Mmp19 (34-fold), Mmp2 (15-fold), Myc (7-fold) and Tnf (6-fold). The highly downregulated genes included Bdnf (2.5-fold), Fos (3.3-fold) and Jun (5-fold).

### Changes in gene expression following rTMS

We conducted a one-way ANOVA to determine if rTMS induced changes in gene expression after MCAO and if they depend on the rTMS protocol. Similar to the previous analyses, we initially assessed the data for outliers by inspecting the boxplots and assessed normality with the Shapiro-Wilk test. Only the expression of 8 genes was not normally distributed in one of the groups (Shapiro-Wilk test, *p*<0.05). The expression of all other genes had normal distributions in all groups. Table 2 shows the homogeneity of variances for each gene (Levene's test of homogeneity of variance). Because the assumption of homogeneity of variances was violated for majority gene expression, a one-way Welch ANOVA was conducted to determine if the expression of genes differed between the groups. This test was followed by a Games-Howell post-hoc test for multiple comparisons (see Table 2, group difference column).

**Table 2:** Fold-changes in mRNAs (RQs) of all genes that showed expression on the microarray after two weeks of rTMS using the MCAO group as a calibrator. MCAO, 1, 5, and Sh in the group significance column indicate significant difference to MCAO, 1Hz, 5 Hz, and ShSTIM groups, respectively.

Gene Symbol	1 Hz	5 Hz	cTBS	cTBS Gr. Sig. Dif.	iTBS	iTBS Gr.Sig. Diff.	Variance	F value	df1	df2	Sig.
Actb	0.81	1.09	1.89		1.98		0.001	2.608	5	18.627	.059
Adcy1	0.72	1.11	1.43		2.28		0.001	2.048	5	18.497	.119
Adcy8	1.15	1.12	1.81		2.87	MCAO Sh	0.001	2.902	5	18.204	.043
Adm	1.35	0.83	2.05		1.49		0.011	1.050	5	18.624	.418
Adrb1	0.94	1.30	1.71		2.49		0.021	1.809	5	18.047	.162
Ak1	0.80	0.93	1.41		2.72		0.006	2.331	5	17.516	.086
Angpt1	1.00	0.81	1.98		2.19		0.001	1.237	5	17.871	.333
Angpt2	1.22	1.02	1.98		1.52		0.001	.595	5	18.244	.704
Apoe	0.78	0.80	1.09		1.63		0.001	2.214	5	18.715	.096
App	0.68	0.98	1.70		2.60	MCAO 1 5 Sh	0.001	4.777	5	17.729	.006
Arrb1	0.75	0.94	1.56		2.97	MCAO 1 Sh	0.001	3.921	5	17.865	.014
Arrb2	0.80	1.03	1.51		1.98		0.043	2.260	5	17.689	.093
Atf3	0.83	0.72	1.59		1.38		0.001	.958	5	18.072	.469
Bag3	1.03	0.90	1.96		2.25		0.001	1.905	5	18.631	.142
Bai1	0.60	0.91	1.71		2.42	MCAO 1 5 Sh	0.001	4.326	5	17.644	.010
Bdnf	1.43	2.62	3.17		5.82	MCAO 1 5 Sh	0.001	5.490	5	18.274	.003
Btg2	0.76	0.71	1.12		1.08		0.001	2.051	5	18.807	.117
Car11	0.77	0.97	1.09		2.41		0.001	1.896	5	18.153	.145
Cck	0.71	0.82	1.49		3.17		0.003	2.377	5	17.907	.080
Cdk5	0.67	0.78	1.58		2.80	MCAO 1 5 Sh	0.001	4.499	5	17.762	.008
Cited2	0.92	1.16	1.60		2.46	MCAO 1 5 Sh	0.001	3.003	5	17.933	.038
Clock	0.99	1.05	1.90		3.13		0.001	1.905	5	18.091	.143
Cnr1	0.84	0.82	1.63		3.03	1 5 Sh	0.001	3.471	5	18.098	.023
Creb1	0.86	1.05	2.20		3.25	MCAO 1 5 Sh	0.001	4.395	5	18.228	.008
Cryab	1.10	1.16	2.15		3.05		0.001	2.539	5	18.311	.065
Csnk1e	0.66	0.94	1.79		2.57	MCAO 1 5 Sh	0.001	7.680	5	17.957	.001
Cxcl12	1.15	0.85	1.68		1.75		0.006	1.046	5	18.772	.420
Cxcr4	0.74	0.88	2.04		1.57		0.001	1.440	5	18.543	.257
Dagla	0.63	0.88	1.93		2.94	MCAO 1 5 Sh	0.001	4.318	5	17.966	.009
Dbh	1.03	1.16	1.78		2.17	MCAO Sh	0.022	3.260	5	18.871	.027
Ddit4	1.18	0.80	1.29		1.62		0.003	1.707	5	18.063	.184
Dnaja1	1.06	1.04	1.81		3.66	MCAO 1 5 Sh	0.001	2.767	5	18.302	.050
Drd2	0.83	1.00	3.23		6.78	MCAO 1 5 Sh	0.001	3.328	5	18.093	.026
Dusp1	0.62	0.84	1.43		2.76	MCAO 1 5 Sh	0.001	5.989	5	18.358	.002
Egr1	0.82	1.20	2.10		3.17	MCAO 1 Sh	0.001	3.807	5	18.772	.015
Eng	0.70	1.10	1.93		1.29		0.003	1.555	5	18.305	.222
Epn2	1.21	1.12	1.82		3.38	MCAO Sh	0.001	3.045	5	18.090	.036
Faah	0.92	0.95	2.23		4.07	MCAO 1 5 Sh	0.004	4.819	5	18.256	.006
Fgf2	0.93	1.03	1.63		2.34		0.005	1.988	5	18.331	.129
Fos	1.73	1.73	3.78	MCAO 1 5 Sh	3.83	MCAO 1 5 Sh	0.001	11.754	5	18.411	.001
Gabbr1	0.68	0.88	2.34	1 5	3.37	MCAO 1 5 Sh	0.001	8.054	5	17.953	.001
Gad1	0.65	0.83	1.26		2.55	MCAO 1 5	0.001	2.871	5	17.762	.045
Gad2	0.69	1.02	1.84		2.92	MCAO 1 5 Sh	0.001	3.340	5	18.844	.025
Gadd45b	1.08	0.90	1.68		2.16	MCAO Sh	0.001	2.902	5	18.693	.042
Gapdh	0.88	1.00	1.63		3.13		0.001	2.135	5	18.251	.107
Gfap	0.92	0.91	1.81		1.87		0.001	1.014	5	18.364	.438
Gls	0.58	0.85	1.34		3.35	MCAO 1 5 Sh	0.002	3.552	5	18.310	.020
Gnmt	0.64	0.77	1.11		3.45	MCAO 1 5 C Sh	0.001	4.819	5	17.912	.006
Gpx1	0.75	0.93	1.90		1.76		0.017	1.855	5	18.496	.151
Gpx2	1.12	1.30	4.25		2.44		0.002	.756	5	18.168	.593
Gria1	0.84	0.98	1.99		3.06	MCAO 1 5 Sh	0.001	4.960	5	18.035	.005
Gria2	0.86	1.20	2.12		3.16	MCAO 1 5 Sh	0.001	5.981	5	18.183	.002
Gria3	0.96	0.92	1.47		2.51	MCAO 1 5 Sh	0.002	3.095	5	18.325	.034
Gria4	0.97	1.03	1.55		3.19	MCAO 1 5 Sh	0.008	3.273	5	18.670	.027
Grin2a	0.65	0.95	1.87		2.91	MCAO 1 5 Sh	0.001	3.496	5	18.141	.022
Grin2c	1.18	0.99	1.80	MCAO 5 Sh	3.27	MCAO 1 5 Sh	0.001	5.490	5	18.153	.003
Hif1a	0.99	1.00	1.51		2.76		0.001	1.634	5	18.180	.201
Hmox1	0.53	0.76	2.98		0.88		0.002	2.247	5	17.712	.095
Hspa4	0.65	0.83	2.13	1 5	2.69	MCAO 1 5 Sh	0.001	7.314	5	18.265	.001
Hspd1	0.76	0.86	1.65		1.92	MCAO 1 5 Sh	0.001	3.841	5	18.581	.015
Hsph1	0.72	0.97	1.48		2.68	MCAO 1 5 Sh	0.001	3.491	5	18.539	.021
Irf1	0.78	0.77	1.96		1.59		0.001	1.337	5	18.059	.294
Jun	0.91	0.91	1.40		2.16	MCAO 1 5 Sh	0.002	2.611	5	18.717	.050
Junb	0.79	0.86	1.65		1.62	1 5	0.001	3.717	5	16.954	.019
LOC246295	0.88	0.96	2.36		3.47	MCAO 1 5 Sh	0.010	6.647	5	18.162	.001
Mapk1	0.56	1.10	3.74		4.51	MCAO 1	0.001	4.210	5	17.927	.010
Mgll	0.70	0.96	1.33		2.61	MCAO 1 5 Sh	0.004	3.509	5	18.487	.021
Mmp19	0.65	0.84	1.86	5	1.35		0.001	2.797	5	17.948	.049
Mmp2	0.93	1.01	1.97		1.32		0.001	.846	5	18.736	.534
Mmp3	0.64	1.01	1.09		1.58		0.009	1.589	5	18.606	.212
Mmp9	0.77	1.02	1.77		2.81	MCAO Sh	0.001	4.087	5	18.531	.011
Myc	1.02	1.13	1.35		1.84		0.009	.612	5	18.445	.692
Napepld	0.75	1.19	1.59		2.77	MCAO 1 5 Sh	0.005	3.529	5	17.677	.022
Nog	0.65	0.85	1.76		2.05	MCAO 1 5	0.001	3.928	5	17.920	.014
Nos1	0.86	0.84	2.28		3.05	MCAO 1 5 Sh	0.001	3.852	5	18.218	.015
Nptx2	0.77	1.11	1.88		4.14		0.002	2.142	5	18.198	.106
Per3	0.89	0.87	1.42		2.37	MCAO 1 5	0.006	3.046	5	17.845	.037
Plat	0.67	0.80	1.97	1	1.36		0.001	4.281	5	18.757	.009
Pxmp4	0.77	0.89	1.67		2.70	MCAO 1 5 Sh	0.002	3.997	5	18.550	.012
Rdx	1.06	1.08	1.83		3.03		0.001	2.255	5	18.160	.093
Slc3a1	1.31	1.44	3.37		4.40	MCAO 1 5 Sh	0.001	3.319	5	18.242	.026
Tacr1	0.87	0.85	2.43		3.22		0.001	2.406	5	18.727	.076
Tgfa	0.97	0.96	2.17		3.33		0.001	2.599	5	18.655	.060
Tnf	0.29 ^S,C,I^	0.68	2.27		2.06	MCAO 1 5 Sh	0.001	8.515	5	17.045	.001
Trpv1	1.52	0.97	2.11	5	3.87	MCAO 1 5 Sh	0.001	6.265	5	18.186	.002
Tubb5	0.76	1.04	1.68		2.43	MCAO 1 5 Sh	0.001	4.506	5	17.862	.008
Vegfa	1.15	1.17	1.94		3.28	MCAO 1 5 Sh	0.001	7.427	5	18.018	.001

Table 2 shows the results of the F statistics. rTMS induced significant upregulation in expression of 52 genes (see also Fig 4B), while none showed significant downregulation. The majority of changes in gene expression were found after iTBS. Here, only the major changes will be summarized in textual form. Amongst the genes involved in angiogenesis, whose expression was upregulated by MCAO alone, only Bai1, a p53-dependent angiogenesis inhibitor, showed further significantly increased expression after iTBS, while other genes were not significantly affected by rTMS, including Angpt1 and Angpt2, Atf3, Cxcl12, Cxcr4, Eng and Fgf2. Interestingly, Vegfa, an angiogenesis activator, was not altered by MCAO but significantly upregulated by iTBS. With the exception of Tnf, none of the genes related to inflammation and injury showed further significant changes following rTMS though they were upregulated by MCAO (Bag3, CryaB, Gpx1, Gpx2, Hmox1, and Irf-1).

Several genes involved in neuroprotection, repair and tissue remodeling showed increased expression after MCAO and were further upregulated by rTMS. Expression of Creb1, Epn2 and Junb increased after iTBS, and Plat increased after cTBS. Genes that showed no change in expression after MCAO but were upregulated after TBS included Mapk1, involved in proliferation and differentiation, and Tubb5, which codes structural cytoskeleton microtubular proteins. Amongst the genes with expression significantly downregulated by MCAO, Bdnf was significantly increased by 5Hz, cTBS and iTBS (a 6-fold increase), while Bai1, Fos and Jun were upregulated only after iTBS.

The majority of genes showing increased expression following rTMS are involved in neurotransmission and plasticity across several neurotransmitter pathways. Among the examined genes related to glutamate pathways, iTBS induced significant upregulation of Gls, which codes for the enzyme directly involved in the presynaptic terminal conversion of glutamine to glutamate. The mRNAs of all four subunits (Gria 1–4) of the glutamate ionotropic AMPA receptor, as well as mRNAs of the glutamate NMDA receptor (Grin2a and 2c), were upregulated after iTBS. Upregulated mRNAs related to GABA signaling included Gabbr1, which codes the component of the heterodimeric G-protein coupled GABA receptor, and Gad1 and Gad2, which encode the glutamic acid decarboxylase responsible for catalyzing GABA biosynthesis. Several genes involved in the endocannabinoid system were also upregulated. The following were all significantly upregulated after iTBS: Dagla, which encodes a diacylglycerol lipase enzyme involved in the generation of anandamide; Faah, which catalyzes degradation of the endocannabinoid class of signaling lipids; and Cnr1, a type 1G protein-coupled cannabinoid receptor activated by the endocannabinoid neurotransmitters like anandamide, and Napepld involved in endocannabinoid biosynthesis. Upregulated genes related to neuronal plasticity and GPCR signaling included the following: App, involved in regulation of synapse formation and plasticity though regulation of Ca^2+^ channels; Arrb1, involved in agonist-mediated desensitization and downregulation of GPCR receptors; and Drd2, a dopamine receptor D2. Finally, after iTBS, the neurotrophin Bdnf and other genes related to Bdnf pathways were upregulated, including Adcy8 involved in the modulation of G protein activity and Gadd45b involved in the regulation of growth.

### QRT-PCR gene expression

Changes in expression, comparable to those detected by the low-density arrays, were confirmed in three genes examined by fast real-time RT-PCR. Increases in expression were seen in Gabbr1 (3.29-fold), Gria2a (4.13-fold) and Bdnf (6.26-fold), respectively. These findings were similar to the fold-changes seen with the low-density array of 3.37, 3.16 and 5.82-fold for these three genes, respectively. The degree of RQ variation between repeated samples from different animals was below 0.5.

## Discussion

The principal findings of this study have demonstrated that rTMS has the potential to influence the expression of genes involved in regulating multiple and diverse pathways and processes, including those involved in the brain response to ischemia. They also show that changes depend on rTMS pattern and frequency with only iTBS inducing significant changes in gene expression. The results confirm and extend earlier suggestions that rTMS in stroke may enhance the overall brain response to ischemic injury. They also suggest that this may be facilitated through activation of diverse neuronal and network level pathways spanning neuroprotection, cellular repair, remodeling and neuronal plasticity.

### MCAO induced lesion and the neurological deficits

The transient MCAO is a widely-used model of stroke because it closely mimics human stroke induced by occlusion of a large artery [44,45]. In the present study, MCAO induced reproducible cortical injury similar in extent and localization to reported data [45–47]. Namely, the pattern of TTC staining and the volume of infarcted brain tissue did not differ significantly between animals 48 hours after MCAO. It should be noted though that in this study TTC staining was performed 48 hours after MCAO. This may have affected the detection of the infarcted area and the volumetry as it was suggested that TTC should not be used beyond 24 hours since the inflammatory cells harbor intact mitochondria [48,49]. Damage to such widespread and functionally diverse brain regions produced significant motor impairment, as evidenced by significant increase in 5-point neurological deficit score (median BS = 3) and significant decrease (by 6.8 points) of 21-point BDS score at 48h in all MCAO groups. The character and the extent of neurological deficits were comparable to previously reported ones [31–33,35].

### Changes in gene expression after MCAO

The number of genes reported in studies examining global gene expression changes after cerebral ischemia varies considerably spanning 150 [50] to 700 genes [42]. Furthermore, regulated genes show large variations in expression depending on postischemic time [41]. It was also shown that gene expression in the rat brain, although similar, differs between cortical regions, with 30 genes found to be enriched in the frontomedial cortex [51,52]. In this study, genes were selected based on their distinct appearance in the frontal cortex [51,52] and level of modulation by ischemia [41,43,53]. Selection was also based on the limited data on genes shown to be regulated by rTMS [38,54,55]. We also included several genes that may show regulation by rTMS, including those involved in regulation of synaptic plasticity. Thus, although we examined fewer genes than traditionally reported, these genes provided broad insight into the effects of rTMS on the ischemic brain.

Stroke alone induced changes in gene expression spanning several functional categories, which were comparable to changes in gene expression reported in earlier studies [41–43,56,57]. For example, several genes related to angiogenesis, a natural defense mechanism set to restore oxygen and nutrient supply after stroke, were significantly upregulated two weeks after MCAO. Other regulated genes included immediate early genes, heat shock proteins, anti-oxidative enzymes, trophic factors, and genes involved in RNA metabolism, inflammation and cell signaling. The expression of Gfap, an established marker of astrocyte activation in brain ischemia, also increased significantly, further suggesting that the developed model was reproducible and comparable to already-established changes in ischemia, providing suitable substrate to explore the effects of rTMS.

### rTMS induced changes in gene expression in stroke

Two weeks of rTMS induced significant increases in expression of a number of genes across several functional categories. The effects of rTMS on genes related to angiogenesis may appear quite limited, as judged by the significant upregulation of only Bai1 and Vegfa, yet they deserve further attention. Namely, although none of the changes were significant, Angpt1 showed a 2-fold change after cTBS and a 2.2-fold change after iTBS, while Angpt2 reached a 2-fold change after cTBS. Other genes in this functional group like Cxcr4 and Fgf2 were upregulated by stroke and further increased after rTMS, but did not reach statistical significance. The fact that the expression of these genes changed in TBS groups, but not in the 1Hz and 5Hz groups, suggest that rTMS may affect angiogenesis-related genes. This finding is partly corroborated by the significantly increased expression of Vegfa after iTBS, which is involved in regulation of Angpt2.

The former findings also apply to genes involved in inflammation and injury. The genes that were upregulated by stroke were not significantly changed by rTMS, except for Tnf, which encodes a multifunctional proinflammatory cytokine known to be involved in the regulation of a wide spectrum of biological processes including cell proliferation, differentiation and apoptosis. Interestingly, although not reaching significance, GFAP expression was 1.8-fold higher after cTBS and iTBS, while there were no changes after 1Hz and 5Hz (0.9-fold change) (see Table 2). Earlier studies showed that acute application of rTMS (25 Hz) induced a profound but transient increase in GFAP [58], while chronic (11 weeks) rTMS had no effect [25]. Current results suggest the opposite effect, as if TBS may increase GFAP expression after two weeks of stimulation in MCAO, while low-rate rTMS (1Hz and 5Hz stimulation) has no effect.

Among the genes related to neuroprotection, cellular repair and remodeling, rTMS significantly influenced expression of several genes, including the immediate early genes Fos, Jun and JunB. In normal healthy animals, Fos expression was upregulated after single exposure to 1 and 10 Hz rTMS [38], as well as after chronic (14 days) exposure of 20 Hz rTMS [54]. In MCAO, chronic (7–28 days) exposure of 0.5 Hz rTMS induced significantly increased expression of Fos [59]. In the present study, Fos increased after TBS, but not after 1 and 5 Hz rTMS, suggesting that the effects may depend on stimulation frequency and/or stimulation pattern. Upregulation of Jun expression, which was also demonstrated earlier after acute rTMS [60], suggests that it may interact with downstream apoptotic mechanisms [43] and potentially the brain’s metabolic response to ischemia [61]. Research has also shown that rTMS may produce increased neuronal survival and a smaller infarcted area after MCAO ischemia-reperfusion [62]; it may also significantly increase expression of the antiapoptotic factor Bcl-2 in the infarcted area, associated with improved learning and memory [63]. Other studies have also suggested antiapoptotic effects of rTMS in subacute cerebral ischemia [24] and bilateral MCAO in rats [23]. Extending these findings, this study reports changes in JunB and other genes related to apoptosis, cellular repair and remodeling (Creb1, Epn2, Mapk1, Plat, and Tubb5), suggesting that rTMS may have more profound effects on apoptosis and remodeling in MCAO. Along the same lines is the finding of upregulation of MMP-9, which in chronic stroke may be involved in neurovascular remodeling, thus promoting brain tissue repair and regeneration during delayed phases after stroke [64]. Finally, GADD45, which is involved in modulation of the cell response to stress was also upregulated by iTBS. Thus, it appears that rTMS may support endogenous mechanisms of neurovascular remodeling in the perilesional cortex as intrinsic (adaptive) repair responses become activated after injury. The main impact of rTMS after stroke was found in the expression of genes related to neurotransmission and plasticity, most of which were not changed by MCAO alone. In the glutamatergic pathway, the effects of rTMS suggest increased presynaptic glutamate production (upregulation of Gls) and NMDA (Gria 1–4) and AMPA (Grin2a and 2c) receptor expression. The ability of rTMS to induce changes in brain activity that last after stimulation relates to changes in synaptic plasticity [65–68], similar to long-term potentiation and depression-like mechanisms [69,70]; thus, it involves modification of activation and activity in the NMDA receptor systems [67,71]. Enhanced functional and structural plasticity after rTMS is also supported by data showing that long-lasting rTMS induces increased glutamatergic synaptic strength in the hippocampus, accompanied by structural remodeling of dendritic spines [72]. The results of changes in gene expression in our study strongly suggest enhanced synaptic plasticity after rTMS in stroke. This possibility is further corroborated by the significant functional recovery at 17d of animals in the iTBS group. In the GABAergic pathway, rTMS significantly upregulated the GABA_B_ receptor (Gabbr1) mRNA, as well as genes involved in the control of GABA biosynthesis (glutamate decarboxylase, Gad1 and Gad2) in the cortical inhibitory interneurons of normal rats, single application of rTMS affects the expression of glutamate decarboxylase isoforms GAD67 and GAD65, activity-dependent proteins which are coded by Gad1 and Gad2 [73]. Furthermore, changes in the expression of these two glutamate decarboxylase isoforms (GAD67 and GAD65) follow complex patterns after rTMS [74], which also depend on the duration of rTMS [75]. While the effects of increased expression of genes related to GABA may be related to its general role in enhancing neuroplasticity and promoting functional recovery, its effects in stroke may require optimal timing, as recently suggested [76].

The data regarding effects of rTMS on the endocannabinoid system are sparse and have not been reported previously, particularly not in stroke. The main effects of rTMS seem to enhance endocannabinoid biosynthesis (upregulation of Dagla, Napepld and Faah) as well as cannabinoid receptor activation and/or synthesis (upregulation of Cnr1). So far, the effects of rTMS on the endocannabinoid system have been demonstrated in relation to depression, effects that seem to be mediated through the Cb1 hippocampal cannabinoid receptor [77]. In stroke, the role of the endocannabinoid system is still poorly understood, but current evidence suggests that its activation may be protective, particularly in relation to reperfusion injury [78].

Finally, the potential of rTMS in promoting neuroprotection and plasticity in stroke is further corroborated by the results that showed a significant increase in expression of genes involved in GPCR signaling (Arrb1, Adcy8 and Bdnf). Bdnf is the most abundant neurotrophin in the CNS and has been associated with numerous functions, such as synaptic plasticity, neurogenesis, motor neuron survival and plastic responses related to motor skill learning and hypoxic ischemic brain injury [79]. Bdnf is also involved in neuronal survival, protecting against neuronal loss while increasing the function of surviving neurons [80]. iTBS induced significant increases in expression of Bdnf (6-fold), whereas earlier studies showed increased Bdnf expression after long-term rTMS in normal rats [81], and after 0.5 Hz rTMS after MCAO [59]. Altogether, our results suggest that rTMS increases expression of genes related to multiple processes that are likely to promote recovery after stroke.

### Changes in gene expression and recovery of behavior deficits depend on the characteristics of the rTMS protocol

Functional effects of rTMS on cortical excitability are determined by the stimulation intensity, frequency and overall stimulation pattern. Studies in human subjects suggest that the direction of changes depend on stimulation frequency and the theta-burst train pattern. High-frequency (> 4 Hz) increases and low-frequency rTMS decreases the excitability [1], whereas an intermittent TBS train enhances, and a continuous TBS train tends to lower the cortical excitability [2]. One of the main aims of this study was to compare the effects of rTMS protocols, which depress (5 Hz and iTBS) or potentiate (1Hz and cTBS) the cortical excitability. This assumes that they may induce disparate effects on gene expression because they most likely differentially affect NMDA-dependent synaptic plasticity. In each protocol, the number of pulses, overall duration of the stimulation and the stimulation intensity were the same. Therefore, the results demonstrate that changes in gene expression in stroke depend primarily on the stimulation pattern (iTBS vs. cTBS) and frequency (1Hz and 5Hz vs. iTBS). Furthermore, they are not related to the direction of the immediate change in cortical excitability. Earlier studies also suggested that long-term 1 Hz stimulation has limited effects on the cortical markers of neuroplasticity [82]. Meanwhile, increased GABA cortical signaling was also reported after long-term 1Hz rTMS, suggesting strengthened cortical inhibition in normal rats [73]. In addition to the stimulation frequency, the absence of effects for chronic 1 HZ stimulation in this study may also relate to altered cortical conditions due to stroke. Conspicuously, several genes showed decreased expression (22 genes with <0.7 fold change) after 1 Hz stimulation, whereas no genes had decreased expression after TBS. Although cTBS appears to be rather ineffective in altering gene expression compared to iTBS, it should be noted that a considerable number of genes were altered after cTBS without reaching significance. The potential difference in the effectiveness between cTBS and iTBS may relate to their cell-type specific effects, particularly in terms of cortical inhibitory interneurons [73], causing differential modulation of their activity [83] and differential activation of pathways in stroke. Differential effects of TBS patterns may also depend on the synaptic connectivity and preferred discharge pattern for these inhibitory neurons [83]. In stroke, this physiological difference may have been further potentiated, rendering certain cortical inhibitory subsystems more or less susceptible to stimulation.

The results of changes in neurologic deficits (BDS) after MCAO and two weeks of rTMS further corroborate the aforementioned conclusions. The improvement in BDS were significantly superior after iTBS stimulation compared to other stimulated groups, strongly arguing for its effectiveness in promoting recovery.

### Methodological aspects and limitations of this study

Brain tissue for gene expression analysis was taken from the cortical areas surrounding the injured core. This method was considered appropriate because the characteristics of the cortical effects in MCAO with reperfusion in rats closely resemble the time course and clinical realities of human stroke. Namely, in MCAO with reperfusion, the ischemic core involves the striatum, which remains densely ischemic, while the overlying cortex undergoes delayed, progressive neuronal death, practically representing ischemic penumbra in this model [84]. Changes in dorsolateral cortex are associated with delayed inflammatory mediators of ischemic cell death and with induction of many hypoxia-induced genes involved in neuroprotection [47]. These genes are induced solely in the cortex, making it the main target for neuroprotective therapies. Because rTMS cannot selectively target the core, penumbra or healthy tissue surrounding the penumbra, it instead affects all of them. Therefore, it appeared warranted to sample changes in gene expression from both the penumbra and healthy surrounding tissue, as they hold restorative potential after stroke.

The use of rTMS in stroke is based on the assumption that it may improve motor function by facilitating the volitional recruitment of corticomotor output neurons, and/or that it may help to re-establish a functional balance between the damaged and undamaged hemisphere. The second assumption, based on the interhemispheric imbalance model [6,85] argues for the use of rTMS to suppress the excitability of the contralesional hemisphere or to boost cortical excitability in the ipsilesional hemisphere, releasing it from excessive interhemispheric inhibition. Although the current study was not designed to explore this hypothesis, it showed the relative ineffectiveness of cTBS in altering gene expression in the lesioned hemisphere. In human chronic stroke, cTBS applied over the non-lesioned primary motor cortex had no effect on the cortical excitability of the motor cortex of the affected hemisphere [86]. However, in acute stroke, cTBS induced a facilitation when applied over the cortical motor hand area of the intact hemisphere [87].

It should also be noted that no significant difference was found in the volume of MCAO-affected brain tissue after rTMS compared to MCAO alone at 17d; there was, however, a trend toward reduction. Earlier studies that examined the spatio-temporal dimensions of ischemic regions showed a similar distribution and size of lesioned areas 7 days after MCAO [47], thus observed changes in volume may be more related to natural evolution of the ischemic injury rather than to rTMS effects.

Certain limitations of the study should be considered when interpreting the results. Excitatory and inhibitory rTMS effects on cortical excitability are primarily based on their immediate effect on the normal brain when probed by MEP. At present, the extent to which they follow the same pattern in a brain stroke remains unclear. Nevertheless, the current results provide insight into the modulation of gene expression by these rTMS protocols, demonstrating that these changes most likely do not depend on the direction of initial changes in excitability. rTMS was performed by standard stimulation coil in this study, causing activation of not only the cortex but also of subcortical structures due to the small size/volume of the rat brain [62,88]. To this ends, an ancillary observation relates to changes in expression of genes in the contralateral hemisphere after rTMS (not shown), which showed no significant changes. Finally, having found changes in expression of more than 50 genes, the study could not establish and quantify the extent to which these genes lead to changes in related proteins; thus, further studies are needed to establish the functional outcomes of changes in gene expression described in this study.

### Conclusions

In summary, the results show that mapping of the gene response may provide significant insight into alteration of molecular pathways and the effects of various rTMS protocols on these pathways in brain ischemia. The results also show the differential effectiveness of various rTMS protocols in inducing changes in gene expression after ischemic-reperfusion brain injury, emphasizing the importance of choice of rTMS protocol when applied over the lesioned stroke hemisphere as therapeutic intervention. Furthermore, they show that the iTBS protocol is the most effective one, affecting a multitude of genes, including those involved in angiogenesis, response to injury, cellular repair, structural remodeling, neuroprotection, neurotransmission and neuronal plasticity. Further studies are needed to underpin the mechanisms of rTMS modulation of gene expression and the eventual role of final products of gene activity in recovery after ischemic brain injury.
